# AlloPool is a deep learning framework that infers protein allostery from molecular dynamics simulations

**DOI:** 10.1371/journal.pbio.3004002

**Published:** 2026-09-21

**Authors:** Matthieu Marfoglia, Miguel A. Pedraza-Joya, Lucas Guirardel, Aisima Chatzi Souleiman, Patrick Barth

**Affiliations:** 1 Interfaculty Institute of Bioengineering, École Polytechnique Fédérale de Lausanne, Lausanne, Switzerland; 2 Ludwig Institute for Cancer Research Lausanne, Lausanne, Switzerland; Stanford University, UNITED STATES OF AMERICA

## Abstract

Recent advances in artificial intelligence have transformed protein structure prediction and design. However, protein function is governed not only by static structures but also by the conformational dynamics that allow proteins to access distinct functional states. Predicting these dynamic transitions, central to many biological processes, remains challenging due to the scarcity of high-resolution experimental data, which limits the training of machine-learning models for dynamic and energetic property prediction. Here, we present AlloPool, a graph neural network (GNN)-based framework that interprets molecular dynamics simulations by iteratively pruning residue–residue interactions to uncover minimal, time-resolved interaction networks that govern protein conformational dynamics and structural responses to chemical or mechanical perturbations, collectively known as allostery. By integrating temporal attention with graph aggregation, AlloPool learns evolving interaction graphs from equilibrium and non-equilibrium molecular dynamics simulations, enabling accurate reconstruction of dynamic trajectories and the interaction networks that drive conformational transitions. Validated across diverse dynamic protein systems, including binding domains, mechanosensors, signaling receptors, and enzymes, AlloPool maps allosteric communication pathways, predicts the effects of ligand binding, mechanical forces, and mutations, discovers transient dynamic states and outperforms existing machine-learning approaches in dynamic trajectory reconstruction. This advance provides a general framework for interpreting protein dynamics with broad implications for drug discovery, synthetic biology, and protein engineering.

## Abstract 2

Protein function depends on dynamic conformational changes that remain difficult to predict despite advances in AI-based structure modeling. This study develops a graph neural network-based framework called AlloPool that accurately extracts allosteric communication networks from molecular dynamics trajectories.

## Introduction

Recent advances in artificial intelligence have transformed protein structure prediction and design [1–4]. However, protein function is governed not only by static structures, but by conformational dynamics that enable proteins to interconvert between distinct functional states [5], respond to external stimuli such as ligand binding or mechanical forces, and transmit information across long distances through allostery [6–11]. Traditionally, dynamic proteins have been conceptualized as molecular switches toggling between discrete conformational states. While useful, this static framework oversimplifies the continuous, multidimensional transitions that underlie allosteric processes [12,13]. Emerging deep learning (DL) approaches have made progress in modeling distributions of discrete protein structural states [14–16]. However, these methods often neglect the dynamical couplings between states and do not explicitly learn the physical interactions that drive conformational change. Consequently, they remain limited in their ability to guide the design of conformationally selective drugs and switchable proteins.

Molecular dynamics (MD) simulations provide a powerful tool to study atomic motions and can now probe timescales relevant to dynamic and allosteric transitions [17–20]. Graph-based computational models, where residues are represented as nodes and their interactions as edges, are frequently combined with MD data to identify long-range residue interactions and map allosteric communication pathways [21]. Techniques such as Perturbation Response Scanning (PRS) and Mutual Information (MI) have shown promise in inferring allosteric pathways [22–24] but suffer key limitations: (1) PRS assumes linear dynamics between perturbations and responses, overlooking non-linear behaviors. (2) MI involves complex post-processing that risks introducing biases [25]. (3) Both methods rely on correlations alone, lacking causal inference to reveal directional signal propagation.

Emerging Machine Learning (ML) approaches, including deep learning and graph neural networks (GNNs), are well-suited to capture complex, non-linear, and temporal relationships and have been applied successfully to dynamic biological networks [26–31]. For instance, Neural relational inference (NRI [30]) has shown promise for identifying allosteric pathways in MD simulations by learning interpretable embeddings of residue interactions [31]. However, NRI relies on static and fully connected interaction graphs, limiting its capacity to model time-dependent conformational changes and learn the interacting residue networks that control protein motions and function. Accurately capturing these fundamental properties requires models that respect the spatial and temporal constraints of physical residue interactions.

To address these challenges, we developed AlloPool, a GNN-based model tailored to interpret complex protein dynamic simulations and predict the residue interaction networks that govern these motions and associated protein functions. Through several innovations (e.g., Temporal Attention Mechanisms, Graph Aggregation Layers), AlloPool overcomes the limitations of static graphs and oversimplified assumptions, excelling in modeling time-dependent conformational changes. We validated AlloPool across diverse dynamic protein systems including protein binding domains, signaling membrane receptors, enzymes and mechanosensors. AlloPool consistently outperformed existing methods in predicting conformational dynamics. It accurately modeled the impact of chemical and mechanical stimuli, as well as amino acid mutations, on protein dynamics and function. Additionally, AlloPool enabled the unsupervised discovery and classification of protein states by detecting changes in temporal interaction patterns. AlloPool represents a significant advancement in the study of protein allostery, providing a robust framework to uncover molecular mechanisms driving allosteric regulation. Beyond advancing our understanding of fundamental biology, it offers a powerful tool for rational drug design, facilitating precise interventions targeting specific protein states. This work not only deepens insights into protein function but also unlocks transformative opportunities in drug discovery, synthetic biology, and bioengineering [32–34].

## Results

### Overview of the model

We developed AlloPool, a graph neural network (GNN)-based framework that captures protein dynamics by learning minimal, time-evolving interaction graphs from molecular dynamics simulation trajectories. The model introduces several key innovations: (1) temporal attention mechanisms, which dynamically adapt interaction graphs over time to capture conformational changes; and (2) graph aggregation layers, which identify critical nodes and edges that mediate conformational transitions and enable long-range allosteric communication.

**Fig 1:**
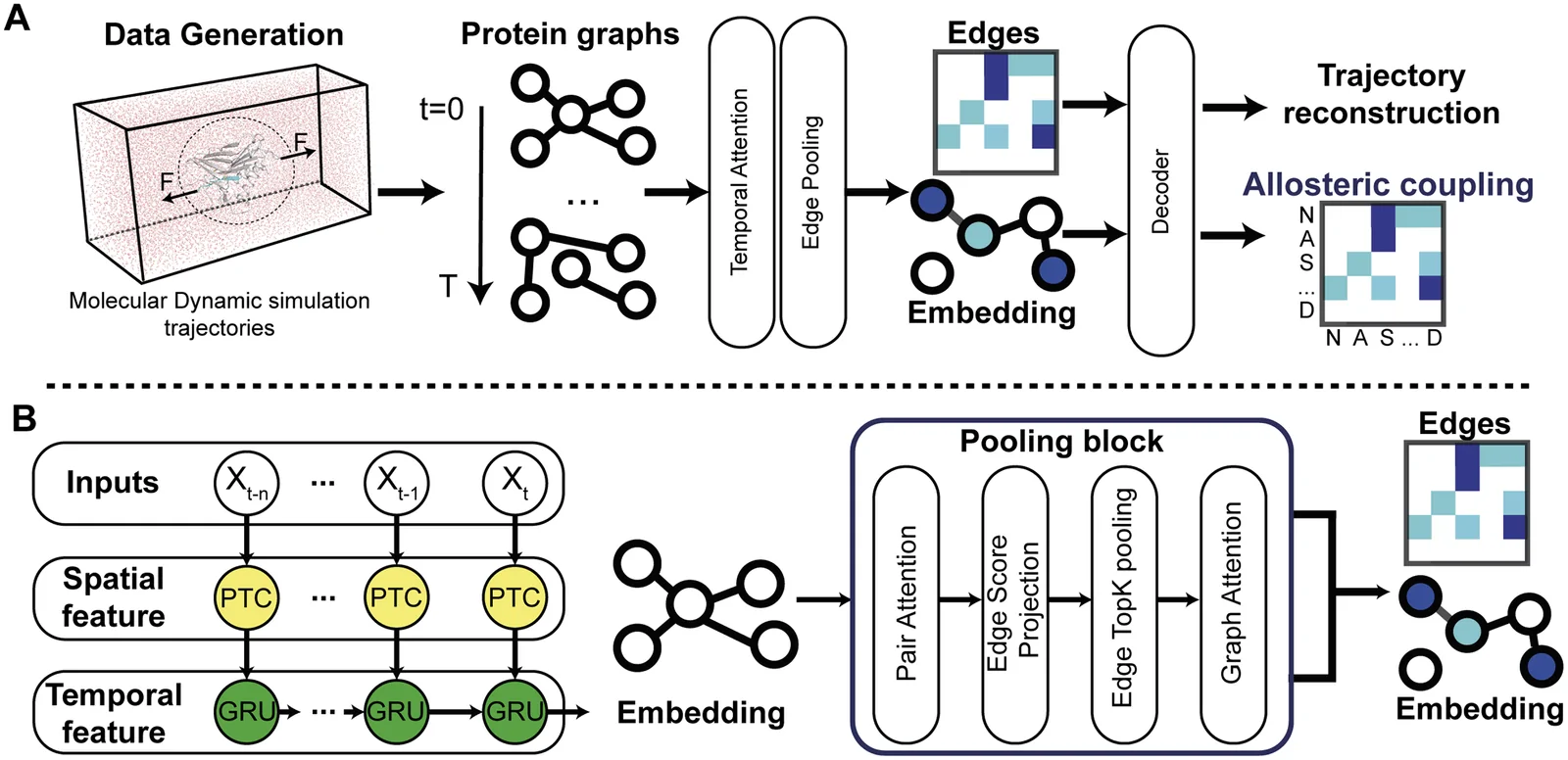
Overview of AlloPool model for learning allosteric interaction networks from dynamic data. **(A)** Simulations are performed to obtain dynamic trajectories of a system (data generation). Each time point of the trajectory is represented by a graph with associated node and edge features. Frames are sampled from the trajectory and used to train the AlloPool model. First, an encoder is trained to select relevant edges from the adjacency matrix generating the latent embedding that depicts the allosteric network of the system. Meanwhile, a decoder is trained to predict the future cartesian displacements (translations) and reconstruct the observed trajectories given the latent representation and the selected allosteric edges. The allosteric process is described as the minimal set of learned edges that enable accurate prediction of the system’s dynamics. **(B)** Temporal graph attention and edge pooling are leveraged in AlloPool for graph embedding and allosteric edges learning. The temporal graph module aggregates the input graphs into a single embedding that is processed by multiple pooling blocks, which iteratively reduces graph connectivity.

The core architecture of AlloPool is designed to learn transition interaction graphs that capture the minimal set of edges driving protein dynamics (Figs 1 and S1). The model processes time-series data from molecular dynamics (MD) or steered molecular dynamics (SMD) simulations, transforming each frame into a graph where nodes represent α-carbons and edges signify spatial interactions between residues. These graphs are fed into an encoder-decoder framework, beginning with a temporal attention layer that condenses the time-series data into a compact representation. The encoder then applies iterative edge-pooling layers, systematically removing edges that do not contribute to the system’s dynamics. This process generates a minimal interaction graph, which is subsequently passed to the decoder for trajectory reconstruction. Through this iterative framework, AlloPool identifies key edges and interaction networks that underlie protein conformational changes, long-range allosteric communication or force propagation.

Principal component analysis (PCA) is applied to the learned interaction graphs to detect shifts in dynamic modes over the simulation trajectories. This analysis reveals significant changes in residue interactions that correspond to conformational transitions, enabling the identification of critical regions within the protein structure involved in dynamic regulation of protein function, allosteric signaling or mechanical force transmission. By reconstructing force propagation pathways, AlloPool can also map how mechanical forces are transmitted through proteins under load, effectively capturing both allosteric and mechanical signaling mechanisms within a unified framework.

### Model architecture

AlloPool is designed to analyze MD trajectories through fixed-length temporal windows. Consequently, long simulations are processed using a sliding-window approach, with representations and predictions generated independently for each window rather than from a single end-to-end pass over the full trajectory. This design enables scalable analysis of arbitrarily long simulations while preserving local temporal information. The model follows a classic architecture of autoencoders to simultaneously learn edges of the protein graph driving its dynamics and the protein’s overall dynamics in an unsupervised manner.

### Graph construction and pre-processing

Our model represents frame data at each time window t as a graph $G_{t}(V,E_{t})$, which represents the protein residues V and their spatial interactions $E_{t}$. Constructing a fully connected graph would be computationally prohibitive and physically unrealistic, as it would include edges between residues that do not interact in the protein structure. Therefore, we decided to define edges based on inter-residue distances: specifically, an edge $(i,j)\in E_{t}$ is included if the distance d(i,j)<15 Å for at least 30% of the frames in the time window [t,t+T−1].

A corresponding set of features is assigned to each of these graphs: The spatial coordinates of the Cα atom, the spatial coordinates of the Cβ atom (hydrogen for glycine), as well as the sine and cosine of backbone dihedral angles. Additionally, we incorporate the displacement of the Cα atom between consecutive frames, which serves as an approximation of the atomic velocity. This results in a total of 13 node features, to which we append a 64-dimensional absolute positional embedding that encodes the residue index within the sequence and the directionality of the amino acid.

We denote $x_{i}^{t}$ the set of features of residue i at frame t, $x^{t}=\{x_{i}^{t}{\}}_{i}$ the set of all residue features at frame t, and $x_{i}=\{x_{i}^{t}{\}}_{t}$ the trajectory of residue i. We also denote $X^{t\to T}=\{x_{i}^{t},...,x_{i}^{t+T}{\}}_{i}$ the trajectory history of all residues starting at time t up to time t+T.

### Encoder: The edge pool module

The general framework of this module aims at acquiring the complex spatial and temporal dependence from the trajectory data and generating an abstract graph representation that aggregates the history of observed timesteps within a given window. This latent graph representation is then processed by an edge pooling module that iteratively reduces the graph connectivity to satisfy our objective function. This encoder is composed of three main sub-modules: a structural attention module, a temporal attention module, and multiple edge pooling modules.

The structural attention module processes the history of residue trajectory data $X^{t\to T}=\{x_{i}^{t},...,x_{i}^{t+T}{\}}_{i}$ and applied a Point Transformer [35], that efficiently processes 3D point clouds, and self-attention to individual graph $G_{t}(V,E_{t})$ to generate new graph representations ${X^{\prime }}^{t\to T}=\{{x^{\prime }}_{i}^{t},...,{x^{\prime }}_{i}^{t+T}{\}}_{i}$ encoding structural features from the observed trajectory data.

The temporal attention module is inspired by previous studies [36] and consists of a GRU model that learns temporal dependence from the trajectory data. We initialize the graph latent representation $h^{\text{0}}$ = 0 and update this latent representation for every ${X^{\prime }}^{t}$ to generate a time-aggregated representation $H^{T}$. Notably, the GRU constructs graph representations by recursively combining the hidden state $h^{t}$at time t−1 with features extracted from the current trajectory frame. An update gate regulates this process by weighting the relative importance of newly acquired information versus previously stored temporal information in the updated hidden state. While capturing sequentially the trajectory information at time t, the model captures the changing trend of structural information and therefore, learns the temporal dependence between isolated graphs $G_{t}\left(V,E_{t}\right)$. This results in a unique graph latent representation that contains both structural and temporal feature of the historical trajectory $G_{latent}(V,E_{t})$.

This graph latent representation $G_{latent}(V,E_{t})$ is processed by multiple edge pooling blocks. Each of these blocks passes the latent graph through a pair attention block that computes score for each edge, and through a graph attention layer, which update node representations, using this procedure:


$$\begin{array}{cc} v_{source},\;v_{target}\; & \leftarrow Linear\left(ReLU\left(Linear\left(H^{T}\right)\right)\right)\\ S_{i,j}\; & \leftarrow MaskedPairAttention(v_{source}+\;v_{target})\;,\;\forall i,j\in E\\ S\text{'}\; & =Sigmoid(\text{Linear}(S_{i,j}))\\ S_{K}^{\prime }\; & ={\text{top}}_{K}(S^{\prime })\\ E_{top}\; & =\{(i,j)\in E\;|\;S{\text{'}}_{i,j}\geq S{\text{'}}_{K}\}\\ \; & \\ H^{T}\; & \leftarrow H^{T}+GraphAttention(H^{T},E_{top},\;S_{K}^{\prime }\;)\\ E\; & \leftarrow E_{top} \end{array}$$

After multiple pooling blocks, the new resulting graph latent representation is $G_{latent}(V,E_{pooled})$.

### Decoder

The task of the decoder is to take this new graph representation, with drastically reduced connectivity, and produce the coordinates ${\hat{x}}_{\theta }^{T}$ for the target portion of the window. The module uses a Multi-Component Spatial-Temporal Graph Convolution network [37] that predicts the 3D spatial coordinates ${xyz}^{t+T}$. Details for the training procedure and analysis of pooled edges and force pathways can be found in the supplementary information.

### AlloPool outperforms state-of-the-art ML models in protein dynamic trajectory reconstruction

To evaluate AlloPool’s performance relative to models utilizing fully connected graphs, we compared AlloPool with the NRI model [31] on both equilibrium and non-equilibrium (e.g., under applied force) simulations of several protein dynamic systems including the peptidyl-prolyl cis/trans isomerase Pin-1, the GPCR autoproteolysis-inducing (GAIN) mechanosensor domain from the adhesion ADGRG1 receptor, the signaling dopamine D2 and the beta 1 adrenergic (B1AR) receptors (S1 Table). During the simulations, the GAIN domain and D2 receptor undergo large conformational changes and sample multiple transitions while the Pin-1 and B1AR simulations sample the dynamics of specific conformational states. We used published simulation datasets for Pin-1 [31]. Three Pin-1 states were selected for the comparison: the apo form (PDB: 1PIN), an agonist-bound state (PDB: 1NMV), and an antagonist-bound state (PDB: 3TDB). Both AlloPool and NRI models were independently trained on each Pin-1 state with parameters defined for each respective model (Methods). All-atom simulations ranging from 1.2 to 2.5 microsecond timescales were used to train individual models for B1AR, D2 and GAIN (Methods).

To compare the models’ abilities to reconstruct protein dynamics, we calculated the standard deviation of the reconstructed trajectories’ Root Mean Square Fluctuation (RMSF) values. To confirm that each model accurately recapitulates the structural dynamics of Pin-1, we also computed the Root Mean Square Deviation (RMSD) between the true and reconstructed trajectories. The results for the RMSD time evolution are presented in S2 Fig, and average RMSD and RMSF in Table 1.

**Table 1:** Comparison of AlloPool and NRI performance in trajectory reconstruction.

System	Pin-1 apo	Pin-1-FFpSPR	Pin-1-pCdc25C	GAIN	D2DR	B1AR
**NRI RMSD**	4.83 Å ± 0.41	1.63 Å ± 0.12	5.72 Å ± 0.32	2.9 Å ± 0.24	2.89 Å ± 0.42	3.12 Å ± 0.18
**NRI VSD**	0.39 Å	0.24 Å	0.91 Å	0.16 Å	0.28 Å	0.31 Å
**AlloPool** **RMSD**	**0.95Å** ± 0.23	**0.93 Å** ± 0.17	**0.82 Å** ± 0.12	**0.89 Å** ± 0.10	**0.89 Å** ± 0.19	**0.64 Å** ± 0.09
**AlloPool** **VSD**	**0.08 Å**	**0.04 Å**	**0.08 Å**	**0.04 Å**	**0.07 Å**	**0.04 Å**
**AlloPool** **RMSF’s Pearson r**	**0.9990**	**0.9878**	**0.9982**	**0.9939**	**0.9975**	**0.9967**

Comparison of average root-mean square deviation ± stderr (RMSD, Å) of α-carbons, value standard deviation (VSD, Å) of root-mean square fluctuations (RMSF) and correlation coefficient of the RMSF. The best model value for these metrics is shown in bold.

Overall, our AlloPool models outperformed the NRI models across these systems. While NRI models were able to replicate trajectory fluctuations as measured by RMSF, we observed a notable variability in RMSD errors, ranging from 1.4 to 5.72 Å across the different protein systems’ trajectories. This variability suggests that NRI models may capture intrinsic system fluctuations but fail to accurately reconstruct the translational displacements observed between frames in the simulation that maintain correct protein topology. Here, we define translations as the displacement in Cartesian space between two simulation frames. AlloPool outperformed the NRI in both fluctuation and structural accuracy categories, achieving subangstrom accuracy in reconstruction RMSD. This performance indicates AlloPool’s effectiveness in generating structurally valid and accurate dynamic transitions, a result attributed to AlloPool’s ability to capture key allosteric interactions across various protein systems and dynamics properties including equilibrium and non-equilibrium simulations.

To evaluate the contribution of AlloPool’s specific architectural components to its prediction accuracy, we conducted ablation tests by systematically removing either the temporal attention or the pair attention modules (S3 Fig). These truncated model variants were trained on GAIN simulations. In the absence of temporal attention, AlloPool did not retained accurate predictions of root-mean-square fluctuations (RMSF); it also failed to correctly predict conformational changes in highly fluctuating regions, increasing the root-mean-square deviation (RMSD) from 0.89 Å (achieved by the full model) to 1.3 Å. Removing the pair attention module had a more pronounced effect, significantly degrading both RMSF and RMSD performance, with the latter reaching 2.1 Å. This was attributed to the removal of long-range information captured by attention, leading to more uniform and less informative edge pooling, which reduced the model’s ability to capture essential interaction patterns.

### Computational advantage of AlloPool

AlloPool utilizes a deep, multi-stage mechanism to arrive at its latent graph, which results in several graph convolution operations dedicated solely to edge scoring and pruning. In contrast, the NRI-MD encoder typically uses a simpler, shallower multi-layer perceptron architecture to compute edge probabilities in a single forward pass. Let N represents the number of amino acid residues (nodes) in the protein system. n the fully connected scenario (NRI-MD), the number of edges E is precisely N(N−1), resulting in an asymptotic scaling of $O\left(N^{\text{2}}\right)$. The computational complexity for calculating the latent edge embeddings is proportional to the number of edges multiplied by the feature dimension d. Therefore, the time and memory complexity for the NRI-MD encoder is strictly bounded by $O\left(N^{\text{2}}d^{\text{2}}\right)$. On the other hand, in AlloPool’s iterative pruning, the initial graph is constructed using a distance cutoff (e.g., 10–14 Å). This reduces the average node degree k of the graph, and the initial number of sparse edges is $E_{init}=k\cdot N$. Thus, the graph size scales linearly with the protein size O(N). During the iterative pruning phase, the Edge-TopK blocks process sequentially smaller graphs. If $E_{m}$ is the number of edges at block m, where $E_{init}>E_{\text{1}}>\ldots >E_{M}$. The complexity of the SageConv layers and the scoring mechanism is proportional to the number of edges present at that specific block. The total computational complexity for AlloPool’s encoder would then be the sum of these sequential operations: $\sum_{\text{m}=\text{1}}^{\text{M}}O\left({\text{E}}_{\text{m}}\cdot {\text{d}}^{\text{2}}\right)$. Since the number of edges strictly decreases, $E_{m}\leq k\cdot N\;$for all blocks, the asymptotic complexity is bounded by: $O\left(M\cdot \;N\cdot d^{\text{2}}\right)$.

In summary, there is an undeniable computational overhead associated with the depth of the AlloPool pooling module. However, the computational efficiency for graph-based architectures is dictated not by the constant depth of the model, but by the asymptotic scaling of the underlying data structure being processed by those layers, in which AlloPool scales better than the NRI baseline.

To illustrate AlloPool’s computational advantages, we performed a direct quantitative comparison of the training and inference efficiency of AlloPool and NRI across three representative molecular systems in our dataset: PDZ, Pin1, and D2DR (S2 Table). In all cases, AlloPool was substantially more efficient than NRI, reducing training and inference times by up to 50-fold as well as memory consumption. These results demonstrate that, in addition to significantly improving trajectory reconstruction accuracy, AlloPool offers considerable gains in computational speed and memory efficiency, enabling both training and inference on molecular systems that are challenging or impractical to analyze with NRI.

### AlloPool accurately predicts large conformational transitions during protein folding and signaling

We evaluated AlloPool’s capacity to learn and reproduce complex dynamic behaviors involving substantial structural changes and transitions between multiple conformational states. The model was trained on non-equilibrium steered MD simulations of the mechanosensing GAIN domain from the adhesion ADGRG1 receptor, the bacterial staphylococcal adhesin SdrG, as well as equilibrium MD simulations capturing transitions between active and inactive signaling states of the dopamine D2 receptor, a GPCR.

### AlloPool predicts force propagation pathways leading to GAIN mechanical unfolding

**Fig 2:**
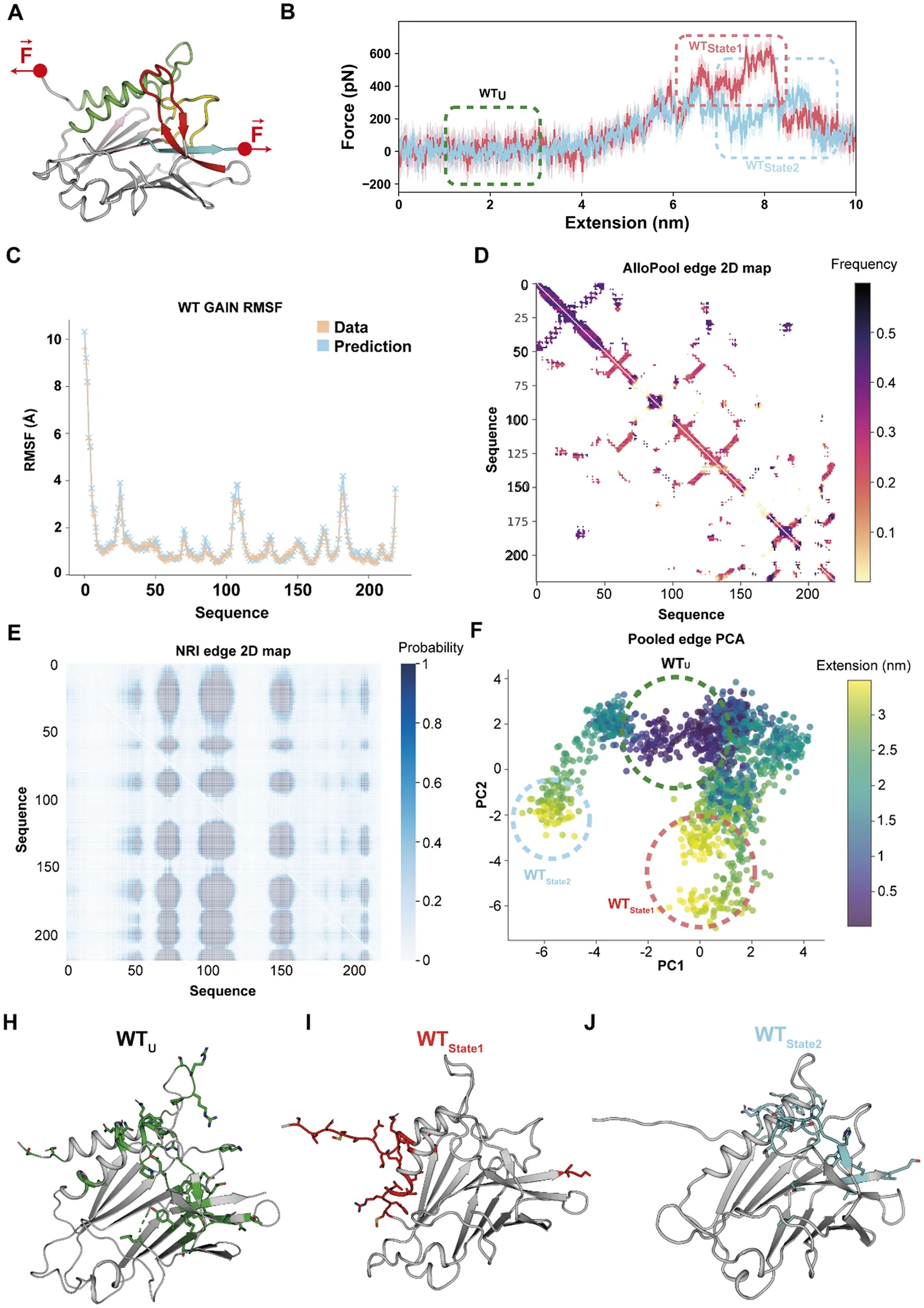
AlloPool identifies multiple transitions to distinct mechanically loaded states. **(A)** Structure of the GAIN domain with the 2 residues onto which the pulling force is applied highlighted by red spheres. **(B)** Representative force-extension curves from SMD trajectory (dark lines) and rolling average (light lines) leading to 2 distinct mechanically loaded states (red, blue). **(C)** Predicted (blue) and observed (orange) RMSF values along the GAIN sequence. **(D, E)** Predicted 2D edge maps by AlloPool (D) and the NRI (E) models. The x-y axes represent the residue index. **(F)** PCA of selected edges represented over the two principal components and colored by time. **(G–I)** Comparison of the directed edges that significantly contribute to the discrimination between the 3 states (G, unloaded state), (H, loaded state 1) and (I, loaded state 2). The underlying data used in this plot is deposited in S1 Data.

The GAIN domain acts as a mechanosensor in adhesion GPCRs. It undergoes mechanical unfolding upon applied mechanical force that releases an agonist peptide activating the receptor [38,39]. To evaluate AlloPool’s ability to predict mechanical unfolding and identify critical allosteric connections (edges) underlying mechanical force propagation in protein structures, we conducted Steered Molecular Dynamics (SMD) simulations on the GAIN domain of ADGRG1 by applying forces at both N- and C-termini with pulling speeds of 0.1 nm/ns and 1 nm/ns (Fig 2A, Methods). The resulting simulation trajectories were used to train AlloPool as per the architecture in Fig 1. Upon pulling, mechanical stress accumulated within the GAIN domain (Fig 2B), leading to conformational changes and the formation of two distinct mechanically-loaded states whose population depended on the pulling rate.

AlloPool was able to reconstruct trajectories with high fidelity, achieving an average RMSD accuracy of 0.89 Å, even in the absence of explicit structural inputs and despite large conformational changes due to partial unfolding of the N-terminal helical subdomain (Table 1). The observed and predicted RMSF describing the dynamic fluctuations for each residue of the GAIN matched also very closely (Fig 2C).

We examined the frequency of edge selections by AlloPool and compared this to the persistence of residue interactions throughout the simulations (S4A and S4B Fig). Persistent interactions showed a binary distribution (S4A Fig), either absent or present, while edge selection frequencies by AlloPool followed a right-skewed distribution (S4B Fig), suggesting that while persistent interactions in any one individual frame may appear unimportant under standard approaches, the model has conversely prioritized the edges critical for the protein’s dynamic state. High-frequency edges aligned with contiguous backbone connections linking adjacent residues but also captured critical tertiary contacts between distal residues, even without direct sidechain information (Fig 2D). Importantly, the interaction network map (Fig 2D) showed asymmetry, illustrating the model’s capacity to create directed edges, thus reflecting a directional flow of allosteric information across the structure. In comparison, the NRI trained on the same dataset only learned diffuse ensemble of edges between subdomains instead of precise residue-resolution allosteric networks (Fig 2E).

To evaluate the functional relevance of the selected edges (Fig 2D) in response to applied force, we reconstructed potential force propagation pathways between the N- and C-termini of the GAIN domain (Methods). Using the rationale described previously [40], we extracted these pathways for both mechanically unloaded and loaded phases of the simulations, prioritizing routes with high average pool frequencies along each path. Remarkably, pathways connecting the residues at the force application sites aligned closely with those identified through correlation-based methods (S5 Fig), indicating that AlloPool effectively identifies edges critical for modulating the GAIN domain’s dynamics under mechanical stress. Additionally, the observed shift in pathway structure between unloaded and loaded states reveals that AlloPool is accounting for structural changes induced by force. These findings suggest that AlloPool successfully identifies force propagation pathways in the GAIN domain, capturing physically-meaningful interactions even when structural alterations occur due to mechanical load.

### AlloPool learns conformational dynamics determinants of GAIN mechanical unfolding

While the trained model successfully captured GAIN conformational dynamics from the SMD simulations, we further examined whether dynamic mode shifts could be identified directly from the learned edges and whether they reflected intrinsic molecular switching properties. PCA on pooled edges (Fig 2F) from the trajectory ensemble revealed that the projected pooled edges correlated with extension, and consequently, force, suggesting that the pooling strategy and learned edges primarily capture the intrinsic dynamics of the GAIN domain rather than variations in SMD trajectories. At low extension (i.e., low accumulated force, Fig 2F, WT_U_), the pooled edges did not differentiate between trajectory replicates. However, at high extension, two distinct transitions emerged, resulting in specific conformational dynamic modes (Fig 2F, WT_State1_ and WT_State2_).

To determine which edges influenced the distribution of unloaded and loaded states, we identified the most discriminatory edges distinguishing WT_U_, WT_State1_ and WT_State2_ and mapped the corresponding nodes onto the GAIN structure (Fig 2G–2I). In WT_U_ (Fig 2G, green sticks), key edges linked helices H1 and H2, helix H2 with loop 10, and β-strands 4–9 with the Stachel. These interactions were stabilized by disulfide bridges within the GPS motif, enhancing the thermodynamic stability of the GAIN domain. In contrast, WT_State1_ was primarily characterized by reinforced connections between helices H1 and H2, as well as β-strand 1, reflecting polar interactions that stabilized the α-domain helices with β-strand 1. Finally, WT_State2_ was distinguished by connections between helix H1, loops 1–7 and 10, and the Stachel, highlighting hydrophobic packing that contributed to mechanical resistance in this loaded conformational state.

These findings illustrate that the model not only captured essential allosteric networks from the simulation trajectories but also uncovered transient mechanically loaded states and distinct dynamic modes governed by different types of interactions, effectively tracking their evolution under mechanical stress. The non-obvious interactions stabilizing the two loaded states, while crucial for GAIN’s mechanical resistance, would be challenging to identify using conventional approaches. This highlights the enhanced temporal and spatial resolution of dynamic modes learned by AlloPool.

### AlloPool predicts force propagation and mechanical unfolding in the adhesin SdrG

To assess the generality of our findings and evaluate whether AlloPool can be broadly applied to mechanosensing proteins, we investigated the prototypical staphylococcal adhesin SdrG. SdrG binds a short peptide from human fibrinogen β, forming an exceptionally strong interaction that anchors the pathogen to the host extracellular matrix. Remarkably, this interaction can withstand forces exceeding 2 nanonewtons [41]. Previous studies have attributed this extreme mechanostability to the redistribution of applied force along the entire fibrinogen peptide, which is tightly confined within the SdrG binding pocket through an extensive hydrogen-bond network. To determine whether AlloPool could capture these mechanistic features, we performed steered molecular dynamics simulations of the SdrG-fibrinogen peptide complex (Methods) and trained our model on the resulting trajectories.

**Fig 3:**
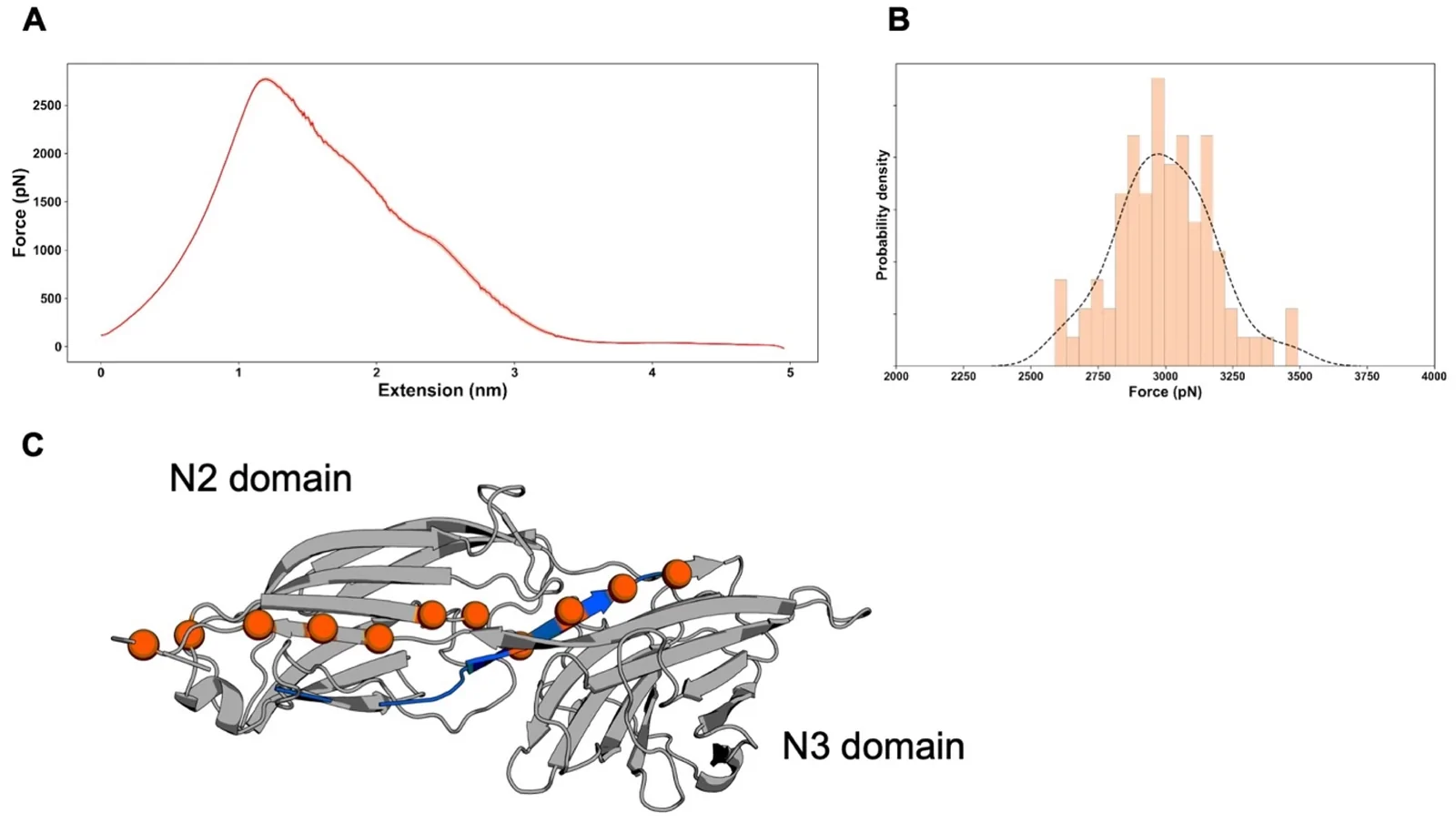
AlloPool predicts force-transmission networks underlying the extreme mechanostability of the SdrG-fibrinogen peptide complex. **(A)** Representative force-extension curves from SMD trajectory of the SdrG:fibrinogen peptide complex. **(B)** Distribution of interface rupture forces across SMD replicates. **(C)** Predicted force propagation pathway mapped onto the SdrG:fibrinogen peptide complex structure. The fibrinogen peptide (blue) is bound to the N2 and N3 domains of the adhesin SdrG (gray). The underlying data used in this plot is deposited in S2 Data.

We observed excellent agreement between the distributions of rupture forces and force-extension curves predicted by AlloPool and those reported previously [41] (Fig 3). Moreover, the force-propagation pathway inferred from AlloPool scores predominantly involves residues within the N2 and N3 domains, confirming that mechanical load is transmitted through the latch strand via a network of adjacent residues.

Together, our results on GAIN domains and staphylococcal adhesins demonstrate that AlloPool provides a general and mechanistically informative framework for dissecting protein mechanical unfolding and force propagation at atomic resolution.

### AlloPool predicts allosteric transition landscapes of GPCR signaling

We next evaluated whether AlloPool could capture the distribution of states sampled during complex allosteric transitions driving membrane receptor signaling. To this end, AlloPool was trained on a cumulative 2-microsecond MD trajectory of the dopamine D2 receptor. These simulations began with the receptor in an active-state conformation, from which both the agonist ligand and the G-protein were removed. Since the apo active-state structure is energetically less stable than the inactive conformation, the receptor spontaneously relaxed toward the inactive state, transitioning through intermediate allosteric states [42].

**Fig 4:**
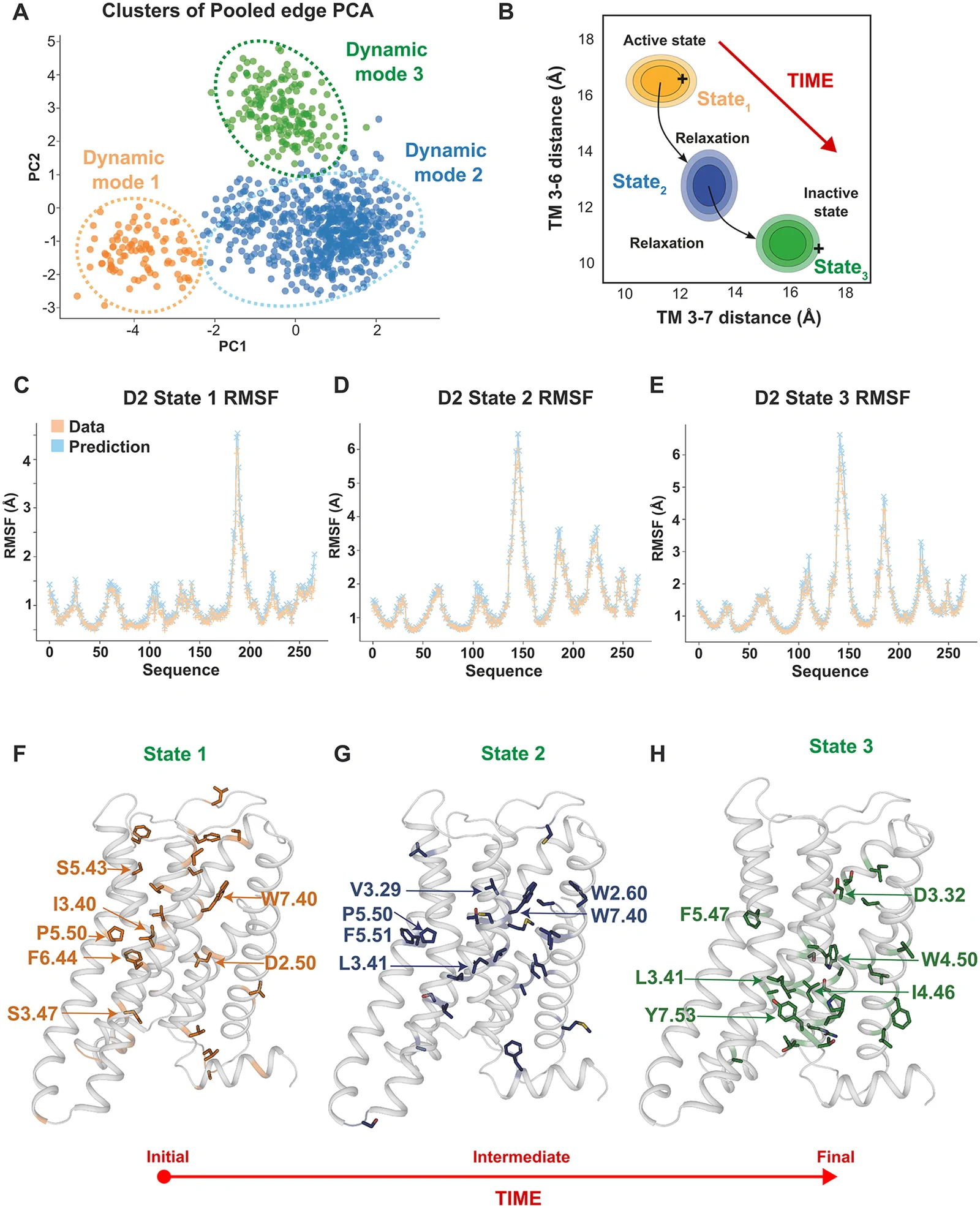
AlloPool predicts allosteric transitions in the dopamine D2 receptor. **(A)** Clustered PCA of selected edges over the two principal components colored by cluster. **(B)** Schematic illustration of the conformational landscape of D2 receptor trajectories showing complete relaxation from active to inactive state, with reference active and inactive structures represented as black + . **(C–E)** Predicted (blue) and observed (orange) RMSF values along the D2 receptor sequence for active (C, state 1), intermediate (D, state 2) and inactive (E, state 3) conformations. **(F–H)** Comparison of the selected edges that significantly discriminate between the 3 dynamic states represented in A (F, State 1; G: state 2; H: state 3). The underlying data used in this plot is deposited in S3 Data.

We assessed whether AlloPool could capture the time evolution of the allosteric interaction networks as the receptor relaxes from the active to the inactive state. Because the pooling network shares weights across all training windows, edge scores are inherently linked to the temporal evolution of the system. Therefore, the learned interaction network is not static but evolves throughout the trajectory, enabling the identification of dynamic changes in residue coupling associated with conformational transitions. Clustering the pooled edges revealed three major dynamic modes, each exhibiting distinct conformational fluctuation patterns (Fig 4A and 4C–4E). When mapped onto a two-dimensional space representing key GPCR activation features; specifically, distances between transmembrane helices 3 and 6 or 3 and 7; these dynamic modes corresponded to three conformational states: the well-characterized inactive and active states, along with an ensemble of intermediate states (Fig 4B).

The dynamic modes associated with inactive and active states displayed distinct sets of pooled edges, which included well-established allosteric residues in GPCRs; e.g., Trp4.50 in the inactive state and the Pro5.50-Ile3.40-Phe6.44 motif in the active state (Fig 4F and 4H). As expected for transient intermediate states, their pooled edges exhibited a mixture of inactive- and active-state features, transitioning from residue critical for either the active or inactive conformations (Fig 4G).

These findings demonstrate that AlloPool can effectively resolve complex allosteric transitions and unbiasedly identify multiple relevant functional and transient states from dynamic data alone.

### AlloPool learns dynamic allosteric networks to predict ligand pharmacology in GPCRs

To evaluate AlloPool’s ability to capture complex allosteric regulation triggered by ligand binding, we benchmarked its performance on the beta-1 adrenergic receptor (B1AR), a classical GPCR with extensively documented ligand pharmacology. Structural studies of GPCRs have identified conserved allosteric residues, or ‘microswitches,’ such as the DRY, PIF, and W-toggle motifs, which drive transitions between inactive and active receptor states in class A GPCRs. GPCR ligands associate with distinct conformational-functional receptor states, classified by their efficacy in activating the receptor and stabilizing its active form. We hypothesized that AlloPool could predict the dynamic allosteric interaction networks associated with different ligand-bound states of B1AR. To test this, we simulated the dynamics of five distinct B1AR-ligand pairs, representing various functional states (antagonist-bound inactive state: B1AR:alp, B1AR:cvd; partial agonist-bound partially active state: B1AR:cyn; agonist-bound partially active state: B1AR:dbt, B1AR:iso). AlloPool was trained on individual trajectories for each ligand-bound simulation, and the resulting allosteric interaction graphs were analyzed.

**Fig 5:**
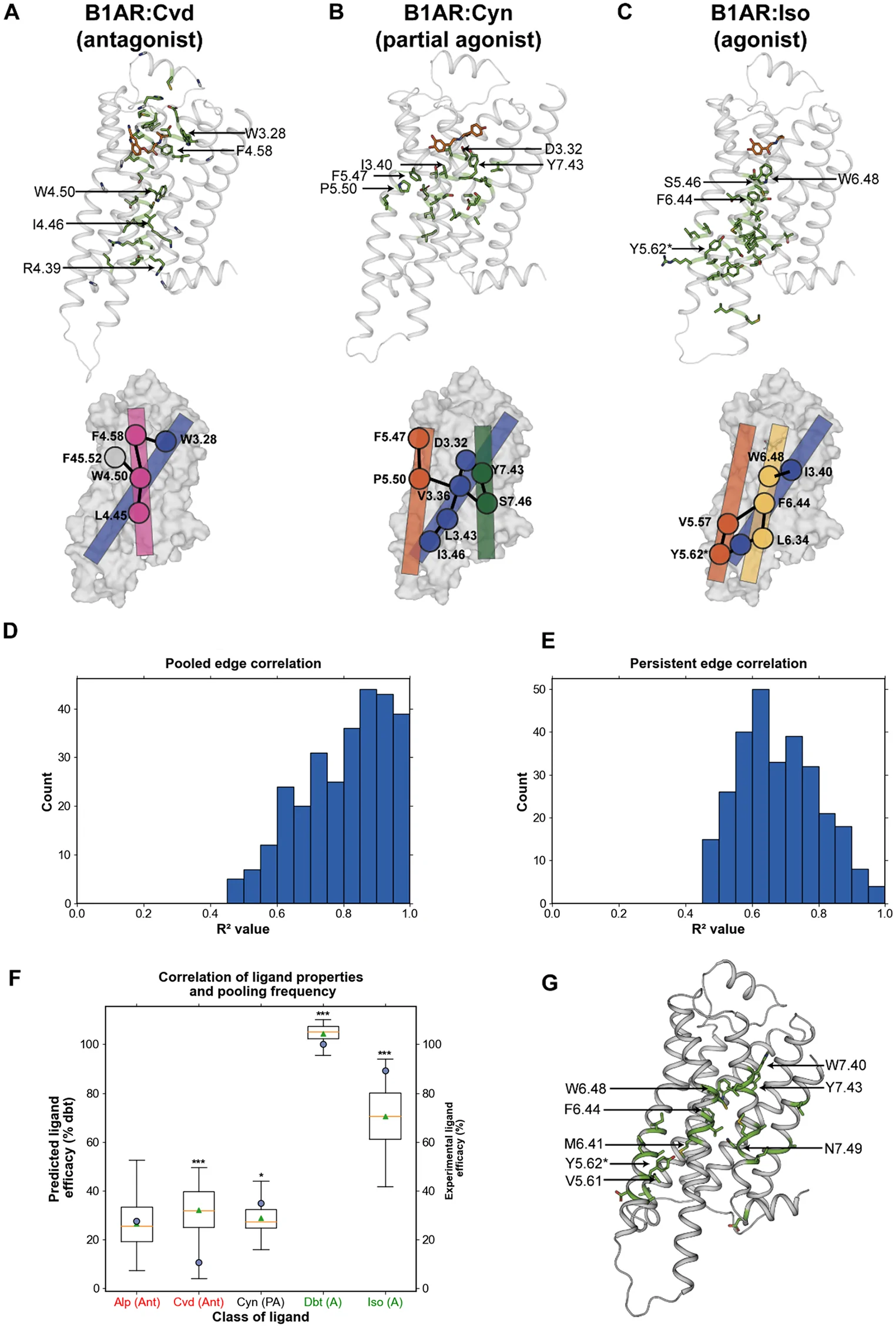
AlloPool learns distinct allosteric coupling of the β1AR receptor in specific functional states. **(A–C)** Structures of the antagonist-bound (A, cyanopindolol, cyn), partial agonist-bound (B, isoprenaline, iso), and agonist-bound (C, dobutamine, dbt) β1AR receptor, with the associated allosteric pathways and key residues represented in the schematics (bottom). The most coupled residues in each ligand-receptor pair are shown as sticks and colored in green. **(D, E)** Histogram showing the distribution of Pearson correlation coefficients for residue based on total contact persistence (E) and total pooled frequency (D) when regressed against ligand efficacy. **(F, G)** Boxplots (orange line: median, green triangle: mean) of predicted ligand efficacy (% dbt) using linear regression of residues with highly correlated pooling frequencies (*R*^2^ > 0.8) compared to experimental ligand efficacy (% dbt) represented as blue circles (Methods). X-axis represents the ligand class (red: antagonist, black: partial agonist, green: agonist). Significant differences from Alp are denoted by stars (*: *p*-value < 0.05; ***: *p*-value < 0.01; n.s.: non-significant) **(F)**, with a structural view on the β1AR structure where correlated residues are represented as green sticks **(G)**. The underlying data used in this plot is deposited in S4 Data.

When trained on B1AR bound to an antagonist, partial agonist, and full agonist, AlloPool identified allosteric interaction networks (i.e., highly connected nodes) occupying distinct receptor regions: TM3-4 for the antagonist-bound state, TM3-5-7 for the partial agonist-bound state, and TM3-6-7 for the full agonist-bound state (Fig 5A–5C). These networks increasingly involve intracellular sites on TM6 and TM7 as ligand efficacy rises, reflecting the large conformational changes in these regions during activation. Notably, the networks from the full agonist state recapitulated key allosteric hubs (e.g., I3.40 and F6.44 from the PIF motif, W6.48 from the toggle motif) and long-range interactions known as hallmarks of GPCR activation. Conversely, the antagonist-bound networks involved TM4 residues, including W4.50, a site associated with negative allosteric modulators stabilizing the inactive state.

We next investigated whether AlloPool’s features could predict ligand efficacy, comparing its performance to classical contact persistency metrics often used in GPCR structural analyses. Persistency metrics showed weak correlations with ligand efficacy (Fig 5D), while AlloPool’s pooled edge features exhibited strong correlations (Fig 5E), with many edges achieving high Pearson correlation coefficients (*R*^2^ > 0.9). Residues with strongly correlated edges that successfully predicted the different ligands’ efficacy (Fig 5F, Methods) were located on TM3, TM5, and TM6, aligning with allosteric sites characteristic of GPCR activation (Fig 5G).

These findings highlight AlloPool’s ability to capture the impact of ligand binding on GPCR allosteric regulation, accurately predicting the dynamic signatures of distinct signaling states. This underscores AlloPool’s potential to elucidate the structural and dynamic mechanisms underpinning ligand-mediated protein functions.

### AlloPool predicts the impact of allosteric mutations on protein binding

**Fig 6:**
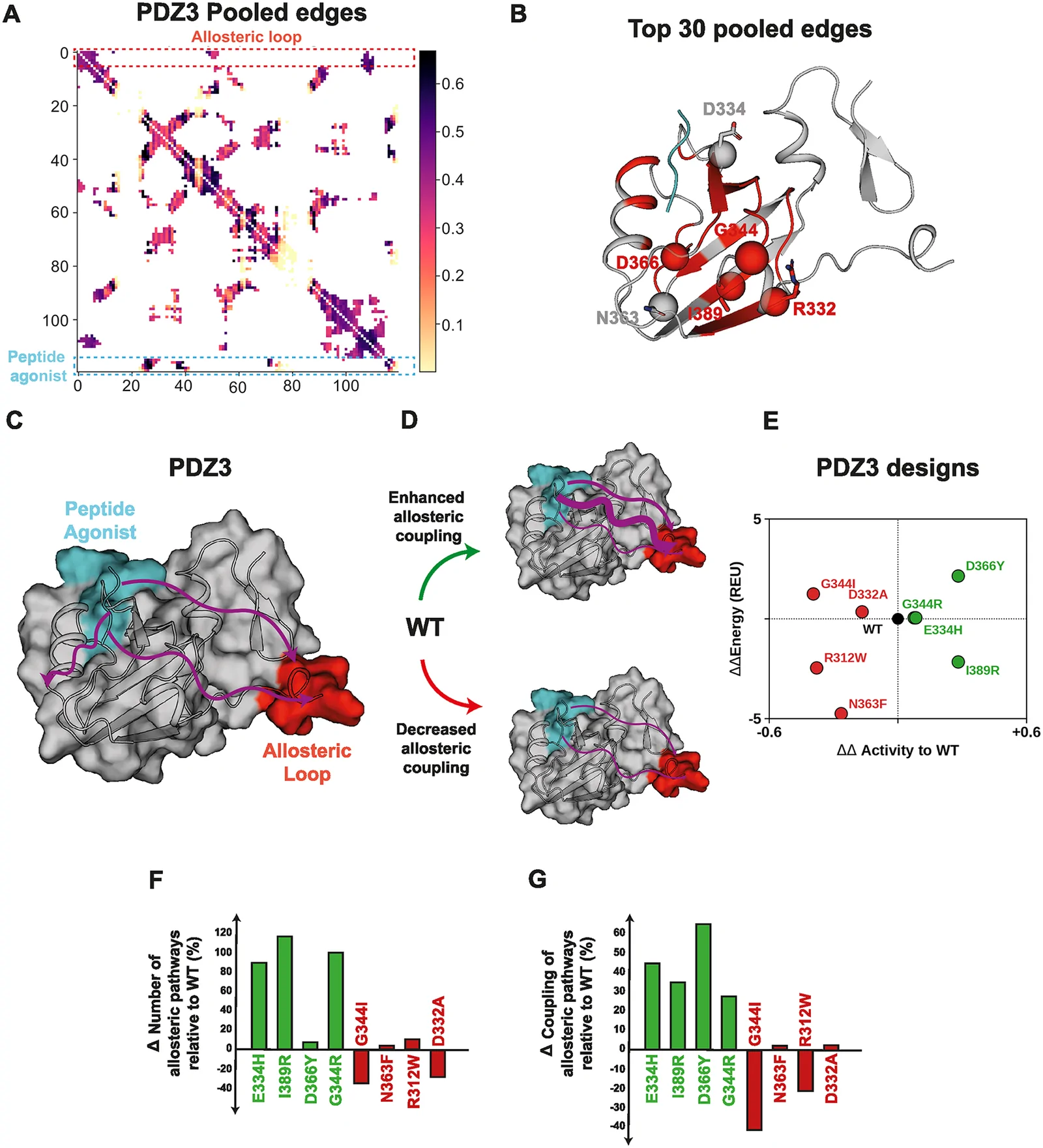
AlloPool predicts the impact of mutations on allosterically regulated protein binding functions. **(A)** Pooled edges of the WT PDZ3 domain. The allosteric loop and the peptide agonist are represented as dashed boxes (red: allosteric loop; blue: peptide agonist). **(B)** Residues involved in the 30 highest coupled edges are shown on the structure (red cartoon). We represented the 7 positions previously reported [45] with strongest gain and loss-of-function mutations as spheres, showing that AlloPool successfully recovers these positions (red spheres: residues with top 10% selected edges by AlloPool). **(C–E)** Expected effect of the mutations on the allosteric coupling between the peptide agonist and the allosteric loop as compared to WT **(C)**, with stabilizing mutations enhancing the allosteric coupling between the peptide agonist and the allosteric loop (D, top) as opposed to destabilizing mutations that disrupt this allosteric coupling (D, bottom). Impact of the mutations on the structural stability of the PDZ structure as calculated using the Rosetta software **(E)**. Energies are reported as Rosetta Energy Units (REU). **(F)** Change in the number of allosteric pathways connecting the peptide agonist to the allosteric loop and with an average pooling frequency along the path of at least 20%. **(G)** Change in average coupling along the allosteric path between the peptide agonist and the allosteric loop for the pathways identified in **(F)**. The underlying data used in this plot is deposited in S5 Data.

Protein association is frequently regulated by long-range conformational changes initiated by ligand binding or mutations at distal allosteric sites, which modulate binding energetics and specificity. Hence, we next assessed AlloPool’s ability to predict the impact of mutations at allosteric sites on protein binding functions. We selected the class of PDZ domains, which are small (~100 amino acid) proteins that typically bind the carboxy-terminal residues of various target proteins and are key components of multidomain scaffolding complexes [43]. Peptide binding to PDZ occurs on alpha helix 2 and beta strand 2 and is allosterically regulated by long-range interactions involving the distal loop region at the N-terminus of the protein [44] (Fig 6A). Mutations at surface positions in these regions have been shown to significantly affect peptide binding [45].

To evaluate this, we simulated the conformational dynamics of the PDZ3 domain bound to an agonist peptide and predicted allosteric interaction networks based on the top pooled edges (Fig 6B). We observed a strong overlap between the residues involved in the 30 highest-coupled edges and those exhibiting significant gain or loss of function upon mutation (Fig 6C). This suggests that the density of pooled edges in the wild-type (WT) protein serves as a strong indicator of allosteric regulation in peptide binding.

To further investigate the effects of mutations on allosteric sites at the protein surface, we simulated the dynamics of eight PDZ variants with experimentally observed large gains or losses of function in peptide binding (Fig 6D). Using these simulations, we constructed putative allosteric pathways linking allosteric sites to peptide-binding sites by connecting nodes with the highest pooled edges (Methods, Fig 6E). We found that, except for one mutant (N363F) exhibiting strong structural destabilization (potentially partially unfolded, Fig 6D), loss-of-function variants displayed either a decreased number of paths or weakened allosteric coupling between allosteric and binding surfaces (Fig 6F–6H). Conversely, gain-of-function variants exhibited stronger coupling or an increased number of pathways.

These findings demonstrate AlloPool’s capability to predict the impact of sequence variations on allosteric regulation of protein binding, highlighting its potential to map functional binding surfaces and uncover the structural underpinnings of allosteric effects in response to mutations.

### AlloPool uncovers dynamic effects of missense mutations invisible to structure prediction

**Fig 7:**
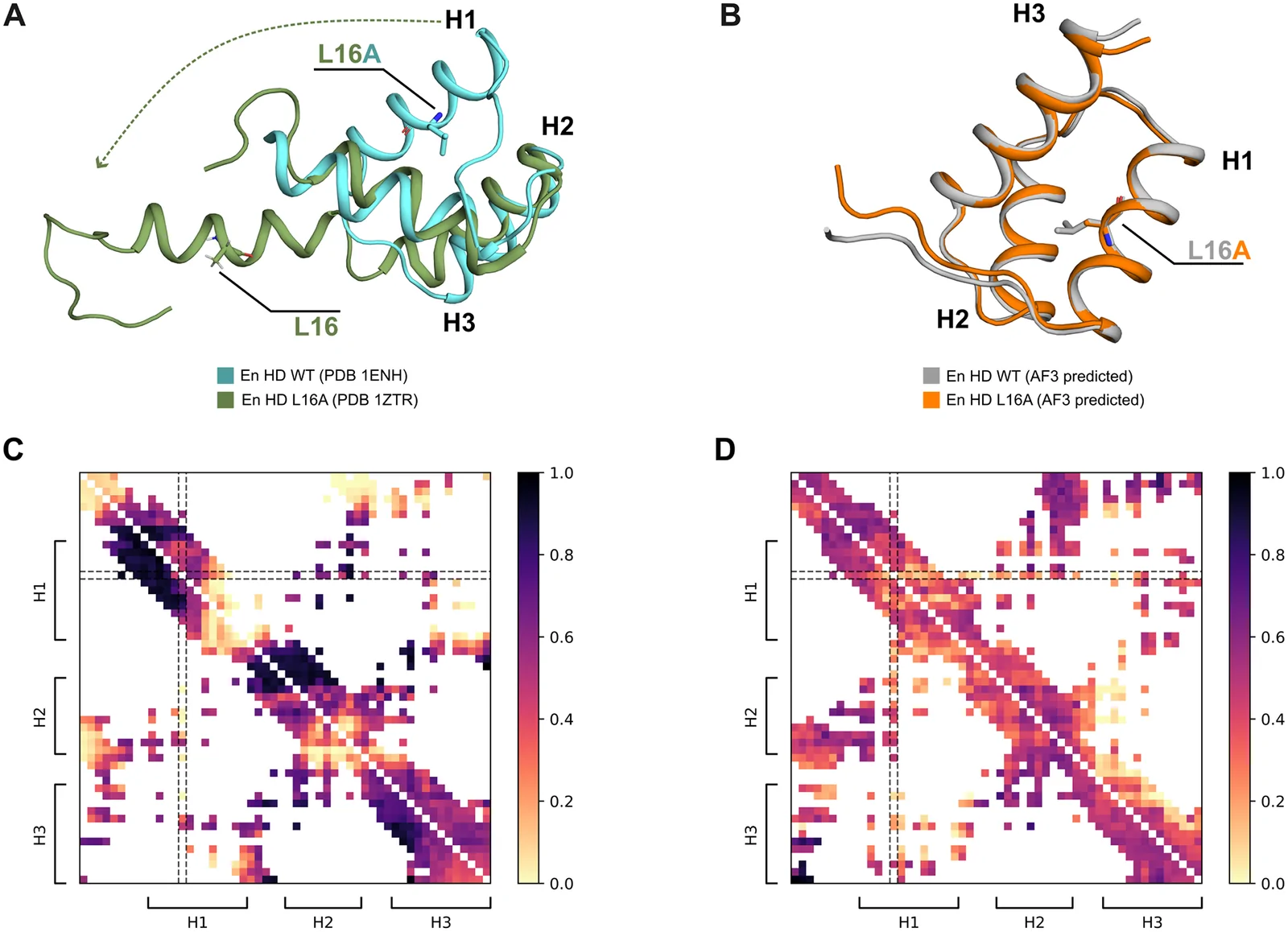
AlloPool pool edge map shifts with missense mutations where structure predictions are not accurate. **(A)** Aligned structures for En-HD WT (PDB 1ENH) and L16A mutant (PDB 1ZTR). The structures are superimposed at the helices 2 and 3 (H2,3). The L16A mutation causes the separation of the H1 helix from the initial helix core **(B)** AlphaFold 3 structure prediction for En-HD WT and L16A. **(C, D)** 2D edge maps predicted by AlloPool colored by pooling frequency for the WT (C) and the L16A mutant **(D)**. The x-y axes correspond to the residue index and were labeled by the helix. The dotted lines indicate the position of the mutated L16 residue. The underlying data used in this plot is deposited in S6 Data.

Despite recent advances in deep learning-based protein structure prediction, accurately capturing the effects of missense mutations remains a major challenge. A striking example is the Engrailed homeodomain from Drosophila melanogaster (PDB ID: 1ENH), a small DNA-binding protein composed of a three-helix bundle. The L16A mutation in helix 1 (H1) disrupts both local interactions and long-range contacts with helices 2 and 3, ultimately leading to the displacement of H1 [46]. Experimental structures of the mutant (PDB ID: 1ZTR) reveal the magnitude of this conformational change, with an RMSD of 17.8 Å relative to the wild-type structure (Fig 7A). In contrast, and consistent with previous reports [47], AlphaFold2 and AlphaFold3 fail to capture the destabilizing effect of the mutation, producing nearly identical wild-type and mutant structures with an RMSD of only 0.339 Å (Fig 7B).

We therefore investigated whether AlloPool could detect the consequences of a missense mutation even when state-of-the-art structure prediction methods could not. To this end, we performed molecular dynamics simulations of the AlphaFold2- and AlphaFold3-predicted L16A mutant structures, accumulating approximately 6 μs of equilibrium sampling, and compared their edge-pooling frequencies to those of the wild-type protein. Because high pooling frequencies reflect strongly coupled residues, whereas lower frequencies indicate weaker correlations and interactions, edge pooling provides a quantitative measure of structural integrity.

Although the simulations were not sufficiently long to reproduce the large-scale conformational rearrangement observed experimentally, AlloPool nevertheless identified the destabilizing effect of the mutation through its learned interaction networks. Specifically, the interaction density between H1 and the remainder of the helical bundle was markedly reduced in the mutant pooled-edge map (Fig 7D) relative to the wild type (Fig 7C). Thus, even though both trajectories remained in a nominally “closed” state, the model detected a substantial weakening of the interaction network that stabilizes the fold.

These results demonstrate that AlloPool can uncover subtle mutational effects encoded in protein dynamics before they manifest as large-scale structural changes. More broadly, they highlight the ability of learned interaction networks to reveal sequence-structure relationships and biophysical properties that are not readily apparent from static structures or direct inspection of molecular dynamics trajectories.

### AlloPool predicts the impact of ligand binding on multi-domain allosteric proteins

We next assessed whether AlloPool could predict the dynamic regulation of multi-domain allosteric proteins triggered by ligand binding. Many proteins consist of multiple domains with the potential for interdomain crosstalk, where the function and activity of one domain can be modulated through the structure and dynamics of another—a phenomenon we refer to as ‘interdomain allostery.’

**Fig 8:**
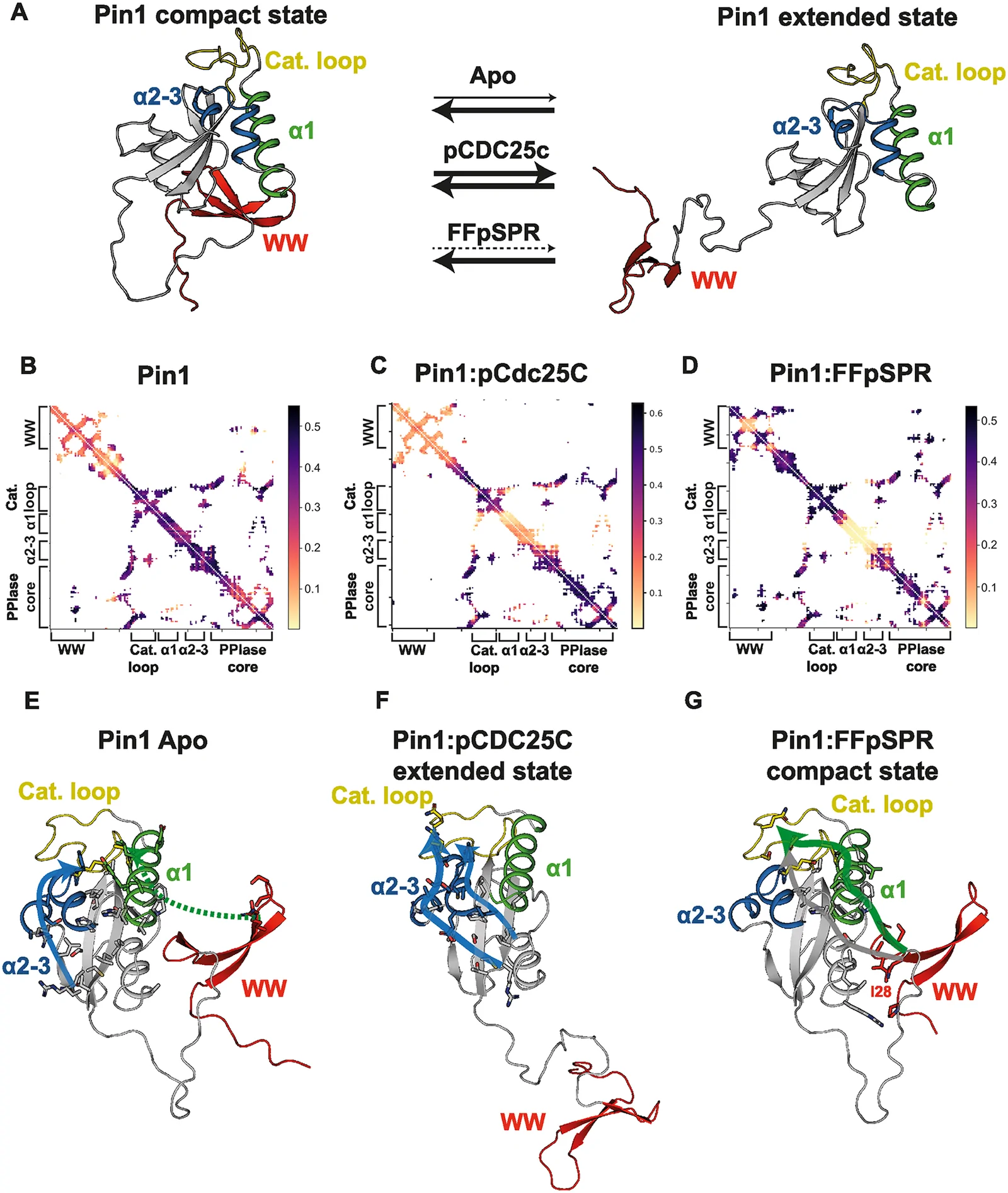
AlloPool predicts the impact of ligand binding on multi-domain allosteric proteins. **(A)** Structural view of Pin1 compact (left) and extended (right) states with key subdomains for Pin1 function represented (yellow: Catalytical loop; blue: helices α2-3, green: helix α1 and red: WW domain). Black arrows (middle) represent the equilibrium between compact and extended state without ligand (apo) or bound to agonist (pDCD25c) and antagonist (FFpSPR). **(B–D)** Predicted 2D edge maps by AlloPool colored by pooling frequency. The x-y axes represent the residue index and has been labeled by key subdomains as in **A. (E–G)** Principal allosteric pathways between the catalytical loop (yellow) and the rest of the Pin1 domain with key residues represented as sticks. Apo state (E) shows strong allosteric pathways connecting the α2-3 domain with the catalytical loop (blue arrow), and between the WW domain, helix α1 and the catalytical loop in the compact state (green dashed arrow). When bound to pCDC25C (F) which favors the extended state, allosteric pathways were only found between the PPIase core (gray), helices α2-3 and the catalytical loop. When bound to antagonist favoring the compact state **(G)**, allosteric pathways connected the WW domain to helix α1 and the catalytical loop. The underlying data used in this plot is deposited in S7 Data.

To evaluate this, we selected the well-characterized Pin1 protein, which undergoes conformational switching between an extended state—where the WW domain is partially unfolded and detached from the PPIase domain—and a compact state, where the WW domain is bound to the rest of the protein [48] (Fig 8A). The compact conformation is favored when Pin1 is either in the apo state or bound to an antagonist (FFpSPR), whereas binding of an agonist (pCDC25c) shifts the equilibrium toward the extended state. AlloPool was trained on multiple MD trajectories corresponding to different ligand-bound states of the protein.

The pooled maps derived from apo and ligand-bound simulations captured the impact of large-scale conformational changes on protein dynamics. In the apo state (Fig 8B), we observed significant coupling between the WW domain and the PPIase core, consistent with the presence of both compact and extended states in the simulation. However, coupling between α1 and the PPIase core was minimal. Most dynamic information, as inferred from pathway analysis, flowed from the PPIase core to the catalytic loop through the α2–3 subdomain.

In the agonist-bound state (Fig 8C), we observed a decrease in coupling between both the WW domain and α1 with the PPIase core. Binding of pCDC25c to the WW domain triggered conformational changes that propagated via the interdomain interface to the catalytic loop, as evidenced by increased coupling between this region and the α2–3 subdomain.

Conversely, in the antagonist-bound state (Fig 8D), the WW domain exhibited increased compactness and folding, leading to enhanced coupling between α1, the PPIase core, and the catalytic loop. We also observed stronger coupling between the WW domain and the PPIase core, but a reduction in coupling between the PPIase core and α2–3.

Remarkably, AlloPool identified pathways connecting nodes with the highest-frequency pooled edges, which closely matched those inferred from NMR spectroscopy [48] (Fig 8E–8G). These findings demonstrate that AlloPool can accurately capture interdomain allostery and ligand-induced conformational changes in multi-domain proteins.

## Discussion

Protein motions are fundamental to function, yet they remain difficult to study, model and analyze. To address this challenge, we developed and validated AlloPool, a deep learning framework that establishes a new standard for interpreting protein dynamics from molecular simulation data. Unlike existing approaches, AlloPool is explicitly designed to learn time-dependent conformational changes and the sparse interaction networks that drive structural transitions. As a result, AlloPool accurately predicts complex dynamic behaviors, including time-resolved fluctuations in allosteric communication, protein binding and folding processes from molecular dynamics trajectories.

AlloPool was rigorously validated across a diverse range of dynamic protein systems, including small soluble domains, membrane receptors, mechanosensors, and multi-domain proteins involved in binding, signaling, mechanical unfolding, and catalysis. Across these systems, AlloPool accurately identified transient conformational states, long-range structural rearrangements, and sparse residue interaction networks governing allosteric transitions, effectively modeling both equilibrium and non-equilibrium dynamics. These capabilities enabled accurate prediction of GPCR ligand pharmacology, identification of allosteric sites, and quantification of allosteric mutations impacting protein binding functions.

Central to AlloPool’s performance are its temporal attention mechanisms and graph aggregation layers, which dynamically evolve protein interaction representations in response to ligand binding and mechanical forces. While most existing methods focus on equilibrium dynamics, many proteins undergo non-equilibrium transitions during folding and function, for which reliable modeling approaches are lacking. AlloPool addresses this gap by accurately predicting non-equilibrium steered molecular dynamics (SMD) trajectories, force-propagation pathways, and key residue networks mediating force transmission during mechanical unfolding of adhesion GPCRs and bacterial adhesins. The directed and asymmetric interaction networks inferred by AlloPool suggest an ability to capture directional force flow, surpassing traditional correlation-based approaches as well as PRS in dynamic contexts. For instance, PRS is based on linear response theory (LRT), which assumes that the response to a perturbation can be inferred from equilibrium fluctuations in the unperturbed state. While this assumption is generally appropriate for systems governed by conformational selection mechanisms, it breaks down in cases involving induced fit or mechanical unfolding, where protein responses become strongly non-linear and are no longer adequately described by the equilibrium ensemble of the resting state. One example in our study where the linear response assumption of PRS is not expected to hold is the mechanical unfolding of the GAIN domain in GPR56. During this process, partial unfolding drives the protein into non-native conformational states characterized by interaction networks that differ substantially from those of the unperturbed structure.

Comparative benchmarking against state-of-the-art NRI models demonstrates AlloPool’s superior performance in RMSD and RMSF metrics, achieved with reduced network complexity through selective edge pooling. PCA and spectral clustering further reveal distinct dynamic modes aligned with force-dependent structural transitions, validating AlloPool’s ability to resolve temporally evolving allosteric dynamics with high precision.

AlloPool’s robust performance across multiple Pin1, GPCRs, and PDZ domain variants highlights its applicability to proteins with multiple functional states. The model enables identification of state-specific allosteric sites, prediction of sequence-dependent allosteric effects, and unsupervised classification of functional states based on interaction network dynamics, providing a powerful framework for modulating protein function.

By combining dynamic edge-pruning GNNs with time-resolved learning, AlloPool captures the inherently dynamic nature of protein function. As a result, its analysis extends beyond static correlation patterns to reveal temporally ordered interaction networks that provide insight into directional signal propagation and causal relationships. This capability is exemplified by the complex conformational transitions of the dopamine D2 receptor, where AlloPool identifies evolving allosteric communication pathways as the receptor relaxes from active to inactive states. Consequently, AlloPool offers a powerful framework for the mechanistic interpretation of allosteric regulation, signal transduction, and the dynamic principles governing protein function.

Despite its strengths, AlloPool has several important limitations. The current model is agnostic to protein sequence and chemistry, as it does not encode amino acid identity and relies exclusively on spatial information extracted from molecular dynamics trajectories. Consequently, AlloPool is not designed to generalize across proteins, and the interaction networks governing conformational dynamics are inherently system-specific, requiring model retraining for each protein and simulation dataset. Addressing these limitations will require substantial architectural advances. Future developments will focus on incorporating richer sequence, structural, and physicochemical information through redesigned encoder–decoder architectures, integration of protein language model embeddings, and higher-resolution representations capable of capturing atomic-level determinants of protein dynamics. Additional improvements may include the incorporation of experimental constraints to enhance predictive accuracy and causal inference frameworks to strengthen the identification of directional communication pathways. Beyond overcoming current limitations, these developments could transform AlloPool into a general framework for deciphering, predicting, and ultimately engineering protein conformational dynamics. Such capabilities would have broad implications for our understanding of allostery and for the rational design of proteins with tailored functions in biotechnology, synthetic biology, and drug discovery.

## Methods

### Molecular dynamics simulations

The data used to train and validate the model was obtained from Molecular Dynamics (MD) simulation of the different system performed with GROMACS 2022.4 [49] with CHARMM36 force field [50] in a NPT ensemble at 310K and 1 bar using a Nosé-Hoover thermostat (independently coupled to protein, solvent and membrane for membrane proteins, with a relaxation time of 1 ps for all three) and Parrinello-Rahman barostat (with semi-entropic coupling at a relaxation time of 5 ps respectively). For membrane proteins, the proteins were inserted into a regular square POPC lipid bilayer using Charmm-Gui [51]. All systems were solvated with a water box with 0.15 M of NA+ and Cl− ions. Equations of motion were integrated with a timestep of 1 fs for the first three steps of equilibration and then 2 fs using the leap-frog algorithm. Each system was minimized using the steepest descent algorithm for 5,000 steps. Then two rounds of equilibration were carried out, the first in an NVT ensemble for 100 ps (50,000 steps with a 2 fs time step.) Position restraints were applied to the protein to allow solvent relaxation around the solute. After minimization and equilibration, 5 independent trajectories of 200 to 400 ns each were run with coordinates being written every 10 ps

AlloPool models were trained on these simulation trajectories by creating samples of five frames regularly spaced every 10 frames. Each window therefore covers a simulation time of 1 ns between its first and last frame. The model is evaluated on a simulation trajectory on which it has not been trained, i.e., a test replicate. Protein structures of individual frames are transformed into distinct graphs with nodes representing residues and edges denoting their connectivity based on the structure adjacency matrix.

For the GAIN domain, SMD simulations were performed using the umbrella sampling method with a pulling speed ranging from 1 Å/ns to 10 Å/ns. In all simulations, the 2 alpha carbons of the N-term and C-term residues were moved harmonically in the desired direction with constant velocity. A distance cutoff of 14 Å was used for short-range non-bonded interactions, whereas electrostatic interactions were computed using the Particle-mesh Ewald method [52] with a cubic interpolation of power 4.

For the bacterial adhesin SdrG, our simulation protocol was analog to that described by Miles [41]. The structure of the adhesin SdrG bound to fibrinogen β is retrieved from the Protein Data Bank (PDB: 1R17) with a resolution of 1.86 Å. The protein was first oriented with the line connecting the Cα of PRO596 and PRO18 lying parallel to the *x* axis. To allow for a sufficient box size during pulling, the box dimensions were set to (58, 7, 7) nm

The MD simulations were performed using GROMACS 2022.4 with the CHARMM36 force field and the TIP3 water model as described above. Once the system was equilibrated, SMD production simulations were performed using Constant Velocity stretching with a pulling speed of 0.25 nm/ns along the *x* axis, and the umbrella sampling protocol with a force constant of 100 kJ mol^−1^ nm^−2^. The reaction coordinate is defined by the Cα atoms of PRO596 and PRO18, with a harmonic restraint applied to PRO596.

### Model training

The model minimizes the RMSD between $x^{T}$ and ${\hat{x}}_{\theta }^{T}$. We compute the RMSD using the Kabsch–Umeyama (KU) algorithm to align the structures prior to loss computation, enabling the loss function to be invariant to rigid body rotation or translation. Formally, the objective function is defined as


$$\begin{array}{c} L(\theta )=\text{R}MSD\left(KU\left({\hat{x}}_{\theta }^{T},x^{T}\right)\right) \end{array}$$

where θ are the trainable parameters of the model h.

The model weights are optimized using the Adam optimizer with an initial learning rate of ${\text{1}\text{0}}^{-\text{4}}$, a weight decay coefficient of 0.1, and a learning rate scheduler decreasing the learning rate by 2 after 100 optimizer steps with no loss decrease. A representative loss curve is included in S6 Fig.

Model selection is performed by tracking the epoch-averaged training loss and checkpointing whenever an epoch achieves a new best value. On improvement, the script saves the new model weights and records auxiliary outputs required for interpretability, attention weights and pooled-edge class assignments, along with metadata describing the run state.

For every system, the validation metrics are calculated on a random trajectory left out during training, with the inference procedure analog to those during training. The results are reported in the main manuscript.

### Pathway calculation

First, we assign each edge a global importance score with its edge selection probability $s_{e}=\left\{ \begin{array}{cc} \frac{S_{e}}{P_{e}} & \text{if }P_{e}>P_{min}\\ \text{0} & \text{if }P_{e}\leq P_{min} \end{array}\right.\;$ where $S_{e}$ (resp. $P_{e}$) is the number of training windows where edge e has been selected (resp. is a persistent edge), and $P_{min}=B/\text{1}\text{0}$ is defined as 10% of the total number of training windows.

Then, we convert edge scores to edge weights $w_{e}=\left\{ \begin{array}{cc} -\text{log}(s_{e}) & \text{if }s_{e}>\text{0}\\ \text{inf} & \text{otherwise} \end{array}\right.\;$. Let $P=(e_{\text{1}},\ldots ,e_{n})$ be a path of length n. We define path score $s_{P}=\text{exp}\left(-\frac{\text{1}}{n}\sum_{e\in P}w_{e}\right)={\left(\prod_{e\in P}s_{e}\right)}^{\frac{\text{1}}{n}}$.

To compute force propagation pathways, we define the shortest paths according to edge weights $w_{e}$ between to sets of residues corresponding to source and target protein regions, and sort them according to path scores.

### Edge PCA and clustering

To identify structural patterns within the pooled edges, we applied a clustering pipeline consisting of dimensionality reduction via PCA followed by spectral clustering.

First, we reconstructed from the pooled edges a residue-level adjacency matrix $A\in R^{N\times \text{N}}$ defined as:


$$\begin{array}{c} A_{i,j}=\left\{ \begin{array}{cc} \text{1} & \text{if edge }e_{i,j}\text{ exists}\\ \text{0} & \text{otherwise} \end{array}\right. \end{array}$$

The ensemble of adjacency matrices $A\in R^{B\times \text{N}\times N}$ (where B is the number of batches extracted from the trajectory data) was projected into a lower-dimensional space using PCA and clustered using the Spectral Clustering algorithm to group adjacency matrices based on their similarity pattern. The optimal number of clusters was selected using the silhouette score analysis.

### Ligand efficacy correlation

To predict ligand efficacy, we employed a linear regression model using pooled edges derived from molecular interaction graphs. We reconstructed adjacency matrices from the pooled edges for every ligand:receptor pair and filter edges with persistence 0.1. A residue is then represented by the vector of its filtered outward edges, a representation used for a Partial least squares regression against experimentally measured ligand efficacy values. To generate predictions, we identified residues that exhibited high correlation (>0.8) with ligand efficacy and predict the distribution of ligand efficacy values from the different ligand:receptor pairs. The statistical significance with respect to Alp is determined by a Wilcoxon signed-rank test.

## Supporting information

S1 Fig Model architecture. Extensive description of the model architecture, from data generation to learning process and evaluation metric.
(EPS)

S2 Fig Time evolution of RMSD for the systems in Table 1. Evolution of the RMSD between AlloPool’s predicted frames and the MD trajectory ground truth for the different systems in Table 1. The average RMSD is represented by the dotted gray line. The data required to reproduce this figure can be found in S8 Data.
(EPS)

S3 Fig Ablation tests. AlloPool performance without Pairwise attention (RMSF **(A)**, Pooled edge map **(C)**); AlloPool performance without Temporal attention (RMSF **(B)**, Pooled edge map **(D)**). The data required to reproduce this figure can be found in S9 Data.
(EPS)

S4 Fig Relative selection frequency of edges. Distribution of edge selection frequency for persistence edges (top) and pooled edges (bottom). The data required to reproduce this figure can be found in S10 Data.
(EPS)

S5 Fig AlloPool predicts force propagation pathways from allosteric interaction networks. Best-scoring force propagation pathways reconstructed from the interaction map learned by AlloPool for the unloaded (left) and loaded (right) states, respectively (top). Force propagation pathways for unloaded (left) and loaded (right) states obtained from classic correlation analysis (bottom).
(EPS)

S6 Fig Representative loss curves for AlloPool training. Loss curve for training AlloPool on the Pin1 trajectories **(A)** and the GAIN domain **(B)** trajectories. Both of these systems exhibited convergence around epoch 90. The data required to reproduce this figure can be found in S11 Data.
(EPS)

S1 Table Summary of MD trajectories used in the manuscript. (XLSX)

S2 Table Computational efficiency comparison between AlloPool and NRI. Computational efficiency metrics during training and inference reported for 3 representative systems PDZ, Pin1 and D2DR. Accuracies of trajectory reconstruction are provided in Table 1.
(XLSX)

S1 Data Supporting Information Data for Fig 2. (Panel_**b**) Rolling average of a single trajectory representative of each loaded state. RMSF (panel_**c**), Pooled edge map (panel_**d**) and Pooled Edge PCA map (panel_**f**) obtained after training AlloPool on a set of GAIN trajectories. Similarly, the NRI edge map (panel_**e**) is obtained after training the NRI-MD using aforementioned trajectories as before. The NRI-MD code is deposited in (https://github.com/juexinwang/NRI-MD).
(ODS)

S2 Data Supporting Information Data for Fig 3. Rolling average of the force as a function of extension for SdRG trajectories (panel_**a**) and maximum force distribution (panel_**b**).
(ODS)

S3 Data Supporting Information Data for Fig 4. PCA coordinates of edges pooled by with the cluster index (panel_**a**) and per-residue RMSF values for each one of the clusters (panel_**c**–**e**).
(ODS)

S4 Data Supporting Information Data for Fig 5. Per-residue vector representation used for linear regression against experimental efficacies for persistent (raw_data*_*panel_**d**) and pooled edges (raw_data*_*panel_**e**). The r2 value from the partial least square regression for persistent (panel_**d**) and pooled edges (panel_**e**). Predictions and r2 value for every selected residue (panel_**f**).
(ODS)

S5 Data Supporting Information Data for Fig 6. Pooled edge map (panel_**a**) obtained after training AlloPool on PDZ3 WT trajectories. Energies calculated with Rosetta for each design (panel_**e**). Number of allosteric pathways and coupling from AlloPool’s learned edges (panel_**f**-**g**).
(ODS)

S6 Data Supporting Information Data for Fig 7. Pooled edge maps for En-HD WT (panel_**c**) and L16A mutant (panel_**d**).
(ODS)

S7 Data Supporting Information Data for Fig 8. Pooled edge maps for Pin1 Apo (panel_**b**), Pin1:pCdc25C (panel_**c**), Pin1:FFpSPR (panel_**d**) obtained from training AlloPool on their respective trajectories.
(ODS)

S8 Data Supporting Information Data for S2 Fig. RMSD between AlloPool’s prediction and ground truth for selected trajectories.
(ODS)

S9 Data Supporting Information Data for S3 Fig. Per residue RMSF (panel_**a**) and pooled edge map (panel_c) for temporal ablation test. Per residue RMSF (panel_**b**) and pooled edge map (panel_d) for pairwise attention ablation test.
(ODS)

S10 Data Supporting Information Data for S4 Fig. Edge frequency for persistent (top_panel) and pooled (bottom_panel) edges.
(ODS)

S11 Data Supporting Information Data for S6 Fig. Representative loss curves for Pin1 apo (panel_**a**) and GAIN WT (panel_**b**).
(ODS)

## Abbreviations

B1AR: beta 1 adrenergic
DL: deep learning
GAIN: GPCR autoproteolysis-inducing
GNN: graph neural network
GNNs: graph neural networks
H1: helix 1
LRT: linear response theory
MD: Molecular dynamics
MI: Mutual Information
ML: Machine Learning
NRI: Neural Relational Inference
PCA: Principal component analysis
PRS: Perturbation Response Scanning
REU: Rosetta Energy Units
RMSD: Root Mean Square Deviation
RMSF: Root Mean Square Fluctuation
SMD: steered molecular dynamics
WT: wild-type
